# Clinical signs and symptoms for degenerative cervical myelopathy: a scoping review of case-control studies to facilitate early diagnosis among healthcare professionals with stakeholder engagement

**DOI:** 10.1038/s41393-025-01065-1

**Published:** 2025-02-26

**Authors:** Suhani Sharma, Alisha Sial, Stone Sima, Ashish Diwan

**Affiliations:** 1 Spine Labs, St George and Sutherland Clinical school, University of New South Wales, Sydney, NSW Australia; 2 Spine Service, Department of Orthopaedic Surgery, St George and Sutherland Clinical School, University of New South Wales, Sydney, NSW Australia; 3 Spinal Surgery, Discipline of Orthopaedic Surgery, School of Medicine, The University of Adelaide, Adelaide, NSW Australia

**Keywords:** Chronic pain, Spinal cord diseases

## Abstract

**Study design:**

Scoping Review.

**Objective:**

Degenerative cervical myelopathy (DCM) is a leading cause of chronic spinal cord dysfunction, with diverse clinical presentations that complicate diagnosis. Therefore, it is important to identify the signs and symptoms of DCM that demonstrate high diagnostic accuracy. This review aims to evaluate the sensitivity and specificity of signs and symptoms in diagnosing DCM.

**Methods:**

Articles up to June 2024 were retrieved from PubMed, EMBASE, and Cochrane databases using search terms like “degenerative cervical myelopathy”, “cervical spondylotic myelopathy”, “sensitivity”, “specificity”, and related signs and symptoms. Studies were screened based on selection criteria assessing the sensitivity and specificity of signs or symptoms using an appropriate control group.

**Results:**

Sixteen studies were included. The most sensitive signs were Tromner sign (93–97%) and hyperreflexia (15–85%). Specific signs included the Babinski sign (93–100%), Tromner sign (79–100%), clonus (96–99%), and inverted supinator sign (78–99%). Neck pain had a sensitivity of 76–94% and specificity of 11–73%. Hand incoordination showed 52% sensitivity and 92% specificity. Altered hand sensation had 76% sensitivity and 90% specificity. Upper extremity weakness had 51–75% sensitivity and 18–95% specificity. Gait imbalance exhibited 56–63% sensitivity and 52–95% specificity.

**Conclusion:**

Sensitive signs like the Tromner sign and hyperreflexia are useful for screening, while specific signs such as Babinski, clonus, and the inverted supinator sign aid in confirmation of DCM. Symptoms like neck pain, hand incoordination, and altered hand sensation should heighten suspicion and guide differential diagnosis. Early and accurate diagnosis using these indicators can improve patient outcomes and reduce diagnostic delays.

## Introduction

**Fig. 1:**
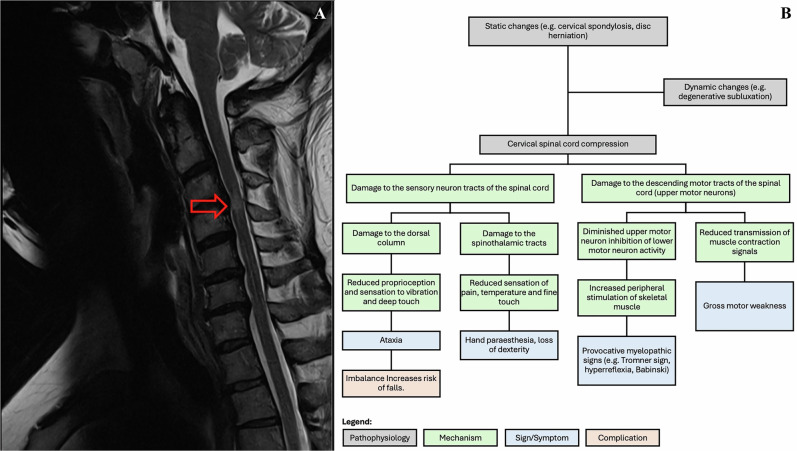
MRI Findings and Pathophysiological Overview of DCM. **A** T2-weighted sagittal MRI of a 45-year-old male with DCM, showing signal cord changes (indicated by red arrow). **B** An overview of the pathophysiological mechanisms, signs, symptoms, and complications associated with cervical spinal cord compression, including both static and dynamic changes.

Degenerative cervical myelopathy (DCM) is a progressive neck region spinal condition first introduced in 2015 and the leading cause of chronic, non-traumatic spinal cord dysfunction worldwide [1, 2]. Despite its prevalence, the diagnosis of DCM remains a significant challenge for practitioners due to its diverse clinical presentations [3, 4]. Consequently, DCM is frequently misdiagnosed, leading to delays in specialist assessment and appropriate management. These diagnostic delays can result in severe consequences, including incomplete post-operative recovery, diminished quality of life, and significant disability, such as the inability to work [5–7]. Research indicates that 20–62% of DCM patients experience clinical deterioration within 3–6 years if surgical intervention was not performed [8]. While surgical treatment is often recommended, patient outcomes depend on the severity of preoperative functional impairment and the duration of symptoms [9–11]. A retrospective study reported that the average time from symptom onset to DCM diagnosis was 2.2 ± 2.3 years [12]. Given the potential for disease progression, prompt diagnosis and timely referral to a specialist are essential to optimize patient outcomes [13]. While DCM’s firm diagnosis requires a clinical radiological concordance (Fig. 1), the importance of DCM suspicion and a sensitivity to considering it as a differential diagnosis in non-specialist situations is an imperative.

To evaluate the clinical utility of signs and symptoms in diagnosing DCM, it is essential to assess their sensitivity and specificity. Sensitivity refers to a clinical sign’s ability to correctly identify patients with DCM, calculated as the proportion of true positives among all patients with the condition. Specificity measures a sign’s effectiveness in correctly identifying patients without DCM, based on the proportion of true negatives among all patients without the condition [14]. These metrics ensure that healthcare professionals make informed decisions, thereby improving patient outcomes through timely and accurate diagnosis.

While systematic reviews and meta-analysis are an important part of translating research to practice, a lack of stakeholder engagement can be a significant obstacle, potentially hindering the implementation of research findings within clinical settings. This scoping review aims to synthesize current research and clinical findings to identify the clinical signs and symptoms of DCM with high diagnostic accuracy, addressing the need for stakeholder engagement in translating research to practice.

## Methods

### Identifying relevant studies

To systematically identify relevant studies on the clinical signs and symptoms of DCM and their diagnostic accuracy, a comprehensive search strategy following the Preferred Reporting Items for Systematic Reviews and Meta-Analysis Extension for Scoping Reviews (PRISMA-ScR) statement was employed across multiple databases, including PubMed, EMBASE, and Cochrane [15]. Publications were retrieved up to June 2024. The search terms were selected to include both MeSH and Emtree terms and free-text keywords, ensuring a broad capture of pertinent literature. The search strategy combined terms for the condition (e.g., “degenerative cervical myelopathy” and “cervical spondylotic myelopathy”) with terms related to clinical signs and symptoms. Diagnostic accuracy terms such as “sensitivity” and “specificity” were incorporated to focus on relevant studies. To exclude irrelevant studies, terms related to imaging techniques (e.g., “MRI” and “CT scan”) were excluded. Only English-language studies were included.

### Eligibility criteria

Table 1 outlines the inclusion and exclusion criteria for selecting the studies included in this review. Table 2 describes the results of individual studies assessing the diagnostic accuracy of clinical signs and symptoms of DCM.

**Table 1:** Inclusion and exclusion criteria.

Characteristic	Inclusion	Exclusion
Population	‐ Patient with cervical myelopathy ‐ Patients 18 years old or more	‐ Patients with spondylosis but do not exhibit myelopathy ‐ Thoracic or lumbar myelopathy ‐ Patients with traumatic cervical spinal cord injury ‐ Patients with tumour or infection
Clinical sign	‐ Tromner’s’ sign ‐ Babinski ‐ Hoffmann ‐ Clonus ‐ Inverted supinator ‐ Hyperreflexia (biceps, triceps, patella, brachioradialis, Achilles) ‐ Tandem gait ‐ Other clinical signs	‐ Studies using imaging modalities to diagnose DCM ‐ Patient (e.g., neck disability index, Visual Analog Scale, SF-36) or clinician (e.g., Nurick grade, mJOA score) reported outcome measures
Symptom	‐ Neck pain ‐ Autonomic dysfunction ‐ Hand numbness ‐ Hand paraesthesia ‐ Upper extremity weakness ‐ Loss of dexterity ‐ Other symptoms	
Outcomes	‐ Sensitivity and specificity of clinical signs and symptoms	‐ Studies reporting only frequencies for clinical signs and symptoms
Study Design	‐ Case – controlled studies with suitable control groups (e.g., individuals with cervical radiculopathy or axial neck pain who do not exhibit myelopathic symptoms)	‐ Review articles ‐ Studies with no control group ‐ Animal studies ‐ Case series, reports

**Table 2:** Results of individual studies assessing the diagnostic accuracy of clinical signs and symptoms in DCM.

Clinical Sign	Authors	Sensitivity (%)	Specificity (%)
Tromner’s sign	Chaiyamongkol et al. [26]	94	79
	Chang et al. [23]	93.5	100
	Soufi et al. [34]	97	35
Babinski	Chaiyamongkol et al. [26]	36	100
	Cook et al. [19]	7	100
	Cook et al. [18]	33	93
	Rhee et al. [24]	13	100
	Harrop et al. [28]	44	Not reported
Hoffmann	Cao et al. [16]	61.6	85.7
	Chaiyamongkol et al. [26]	75	93
	Grijalva et al. [30]	59.2	49.5
	Chang et al. [23]	89.1	100
	Cook et al. [19]	31	73
	Cook et al. [18]	44	74
	Rhee et al. [24]	59	84
	Glaser et al. [29]	58	78
	Soufi et al. [34]	89	41
	Harrop et al. [28]	83	Not reported
Clonus	Cook et al. [19]	11	99
	Cook et al. [18]	7	96
	Rhee et al. [24]	13	100
Inverted supinator sign	Chaiyamongkol et al. [26]	75	86
	Cook et al. [19]	18	99
	Cook et al. [18]	61	78
	Rhee et al. [24]	51	81
Hyperreflexia	Rhee et al. [24]	72	43
	Harrop et al. [28]	85	Not reported
Hyperreflexia of the biceps and triceps	Cook et al. [18]	44	70
Hyperreflexia of the biceps	Cook et al. [19]	18	96
	Rhee et al. [24]	62	49
	Soufi et al. [34]	61	74
Hyperreflexia of the triceps	Rhee et al. [24]	36	78
	Soufi et al. [34]	40	92
Hyperreflexia at the patella	Cook et al. [19]	22	97
	Rhee et al. [24]	33	76
Hyperreflexia at the Achilles	Cook et al. [19]	15	98
	Rhee et al. [24]	26	81
Hyperreflexia at brachioradialis	Rhee et al. [24]	21	89
Suprapatellar reflex	Cook et al. [18]	56	33
Absence of deep tendon reflexes	Philips [33]	13	84
Hand withdrawal reflex	Cook et al. [18]	39	63
Gait deviation	Cook et al. [19]	19	94
Tandem gait	Soufi et al. [34]	66	84
Sensory impairment	Harrop et al. [28]	72	88
	Philips [33]	40	46
Motor impairment	Archer et al. [32]	53	64
	Philips [33]	75	18
Weakness and wasting of deltoid muscles and shoulder girdle	Philips [33]	17	88
Grip release test and grip strength	Kobayashi et al. [20]	88.2	78.1
10 s step test	Machino et al. [31]	92.3	67.8
10 s grip and release test	Machino et al. [31]	77.3	58.8

Symptom	Authors	Sensitivity (%)	Specificity (%)
Neck pain	Soufi et al. [34]	81	73
	Cheung et al. [35]	76	11
	Cook et al. [18]	93	18
Neck stiffness	Hori et al. [36]	11	87
Gait abnormalities/disturbance	Soufi et al. [34]	63	95
	Cook et al. [18]	56	52
Autonomic dysfunction	Soufi et al. [34]	24	95
Loss of dexterity	Cook et al. [18]	72	26
	Soufi et al. [34]	52	92
Hand numbness	Cook et al. [18]	57	67
Hand paraesthesia	Soufi et al. [34]	76	90
Upper extremity weakness	Soufi et al. [34]	51	95
Numbness	Hori et al. [36]	61	60
Pain	Hori et al. [36]	61	60
Tremor	Hori et al. [36]	11	93
Cervical vertigo	Hori et al. [36]	6	87
Hypalgesia	Hori et al. [36]	6	87
Jitteriness	Hori et al. [36]	0	93
Apraxia	Hori et al. [36]	6	93
Difficulty lifting heavy objects	Soufi et al. [34]	75	87
Heightened neck pain when driving or reading	Soufi et al. [34]	Driving: 70 Reading: 71	Driving: 90 Reading: 78

### Data extraction

**Fig. 2:**
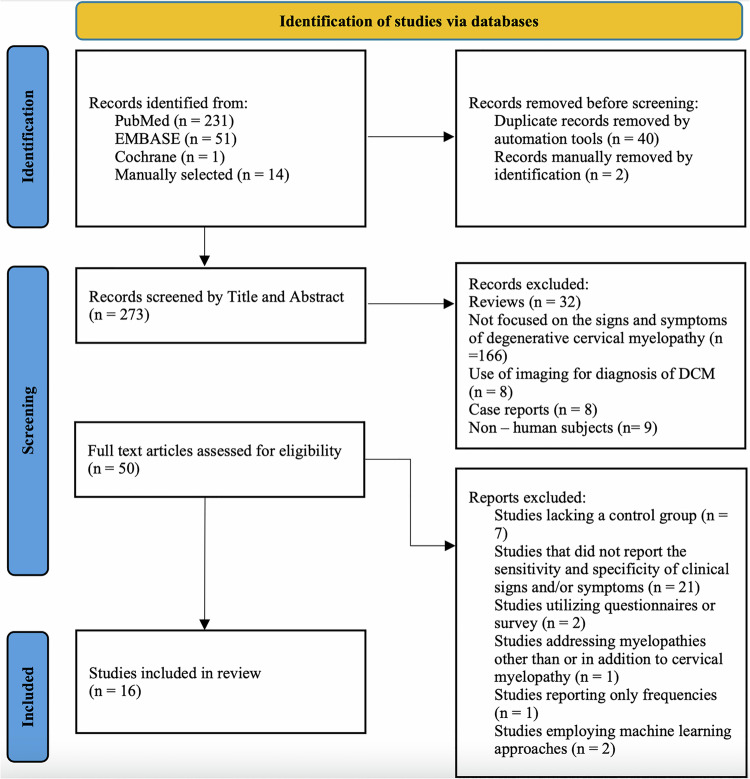
Literature Search Flowchart. Adapted from PRISMA Scoping Review protocol.

Following the search process detailed in Fig. 2, data was extracted from relevant studies included in this scoping review. The key data extracted from each study included the authors, year of publication, title, study design, patient demographics, and the reported symptoms and clinical signs associated with DCM. Additionally, the sensitivity and specificity of each clinical sign and symptom were extracted for inclusion in the results section.

### Collection of codes

Research articles on clinical signs and symptoms for DCM [16–26] were distributed via email to neurosurgeons and spine surgeons (*n* = 11). The collection of codes involved systematically identifying relevant clinical signs and symptoms of DCM from the included studies as detailed in Table 3 to develop evidence-based clinical recommendations for DCM. These codes were refined through discussions among the research team, focusing on clarity, naming conventions, and relationships between codes, as outlined by Mak and Thomas [27].

**Table 3:** Codebook for diagnostic clinical signs and symptoms in DCM.

Code No.	Code Label	Description	Excerpt Text	Comments
1	Clinical Diagnosis	The process of diagnosing degenerative cervical myelopathy based on patient history, physical examination, and imaging studies.	‐ “Despite significant advances in the understanding of the disorder, cervical myelopathy remains a clinical diagnosis. Commonly used criteria in establishing the diagnosis include a history of myelopathic complaints, physical examination demonstrating myelopathic signs, and advanced imaging studies showing correlative compression of the cervical cord.” ‐ “While the presence of these clinical signs is helpful, examination findings may be absent entirely and a diagnosis of DCM can still be made using a combination of patient-reported symptoms and imaging findings.”	Emphasizes the importance of clinical diagnosis despite advancements in imaging.
2	Imaging Confirmation	Any deformation of the cervical spinal cord due to a compressive lesion observed on CT myelogram or MRI.	‐ “Cervical cord compression was defined as any deformation of the spinal cord due to a compressive lesion on CT myelogram or MRI.”	Imaging evidence is critical for confirming cervical cord compression.
3	Absence of Upper Motor Neuron Signs	Diagnosis of DCM can still be made even if upper motor neuron signs are absent.	‐ “The absence of upper motor neuron signs does not rule out a diagnosis of DCM.”	A diagnosis of DCM should not be excluded solely based on the absence of upper motor neuron signs.
4	Sensitivity of Clinical Tests for DCM	The effectiveness of clinical tests in correctly identifying patients with DCM.	‐ “The most sensitive clinical tests for diagnosing DCM are the Tromner sign and hyperreflexia.” ‐ “Most sensitive clinical examination: Tromner and hyperreflexia.”	Tromner sign and hyperreflexia are the most effective tests for initial screening of DCM.
5	Specificity of Clinical Tests for DCM	The effectiveness of clinical tests in correctly identifying patients without DCM.	‐ “The most specific tests are the Babinski, Tromner sign, clonus and inverted supinator sign.” ‐ “Only the inverted supinator sign and the Babinski sign demonstrated significant diagnostic accuracy.” ‐ “Clonus most specific.” ‐ “Most specific clinical examination: Babinski, Tromner, clonus and inverted supinator sign.”	Babinski, Tromner, clonus, and inverted supinator sign are crucial for confirming DCM diagnosis.
6	Diagnostic Accuracy of Clinical Signs in DCM Diagnosis	The reliability of specific clinical signs in diagnosing DCM.	‐ “Only the inverted supinator sign and the Babinski sign demonstrated significant diagnostic accuracy.” ‐ “The most accurate finding to confirm the presence of myelopathy on MRI was the Babinski sign in isolation. Combinations of findings did not improve the diagnostic accuracy of the tests at a rate greater than the standalone test of the Babinski sign.” ‐ “Hoffman may not be present.” ‐ “The Hoffmann sign may modestly increase the predicted probability of cervical spinal cord compression and offer a modest contribution to the overall diagnosis, it is not foolproof for the diagnosis of cervical spinal cord compression.”	Individual signs vary in reliability; combined findings often do not significantly enhance diagnostic accuracy.
7	Clinical Signs and Disease Severity	The relationship between various clinical signs and the severity of DCM.	‐ “There was no definite association between Hoffmann sign, Babinski sign or hyperreflexia and disease severity.	Indicates no clear link between certain clinical signs and disease severity.
8	Clinical Examination Preferences	Preferences for specific clinical signs during examination.	‐ “I always look for the inverted supinator reflex.” ‐ “Hoffmann and clonus”.	Spine surgeons may have preferences for specific signs, such as the inverted supinator reflex, due to their diagnostic value.
9	Sensitive Symptoms for DCM	Symptoms with high sensitivity for diagnosing DCM.	‐ “Hand numbness is frequently reported in DCM patients.” ‐ “Hand paresthesias are a commonly reported symptom in DCM.” ‐ “Loss of dexterity is a sensitive indicator for DCM, particularly in hand function.” ‐ “Neck pain is moderately to highly sensitive for diagnosing DCM, but not specific.” ‐ “Gait abnormalities are often present due to upper motor neuron and proprioceptive dysfunction.”	High sensitivity symptoms should raise suspicion of DCM.
10	Specific Symptoms for DCM	Symptoms with high specificity for diagnosing DCM.	‐ “Autonomic dysfunction is a rare but highly specific symptom for DCM.” ‐ “Bladder dysfunction is uncommon but more frequent than bowel or sexual impairment.”	Specific symptoms, though less sensitive, are still crucial to recognise.

## Results

### Study selection

A total of 283 articles were identified using the literature search strategy, along with 14 articles selected manually (Fig. 2). Following the removal of 40 duplicate records through automation tools and 2 duplicates manually, 273 records remained. These records (*n* = 273) were subsequently screened by title and abstract according to the inclusion and exclusion criteria, resulting in 50 articles being selected for full-text eligibility screening. After a detailed evaluation of each article, 16 articles were deemed eligible for inclusion in the analysis. The complete database search strategy is detailed in Supplementary Table 1.

### Study characteristics

Sample sizes of the included studies ranged from 32–7629. All studies calculated sensitivity and specificity for at least one clinical sign or symptom. Control groups included normal volunteers and patients with cervical spine complaints but no signs of myelopathy or evidence of cord compression. Of the included studies, 62% were prospective and 38% were retrospective. Detailed study characteristics are reported in Supplementary Table 2.

### The sensitivity and specificity of clinical signs of degenerative cervical myelopathy

The study by Rhee et al. [24] found that hyperreflexia had the highest sensitivity (62%), followed by Hoffmann sign (59%) and inverted brachioradialis (51%). In contrast, Babinski and clonus, in conjunction with brachioradialis hyperreflexia, were comparatively less prevalent, with sensitivities recorded at 13 and 21%, respectively.

Two studies published by Cook et al. [18, 19] examined the diagnostic accuracy of various clinical indicators for diagnosing DCM. In the first study, it was demonstrated that the inverted supinator sign had the highest sensitivity (61%), followed by the suprapatellar tendon reflex (56%), while Babinski (93%) and clonus (96%) had the highest specificity. In their second study, the authors sought to develop a prediction model aimed at DCM diagnosis. Based on their results, the combination of gait deviation, Hoffmann, inverted supinator sign, Babinski, and age over 45, were significantly important indicators for DCM diagnosis. Whilst the individual clinical signs had low sensitivity, they exhibited high specificity.

In the retrospective analysis by Harrop et al. [28] sensory loss was the only clinical sign in this study to have both sensitivity and specificity values reported, which were 72 and 88%, respectively. Other signs had only their sensitivity calculated, including gait abnormality (91%), hyperreflexia (85%), Hoffmann sign (83%), lower extremity hyperreflexia (81%), and Babinski sign (44%).

There were two studies that examined the diagnostic value of the Tromner sign specifically in DCM [23, 26]. The sensitivity of this clinical sign ranged from 93–94%, whilst the specificity was 79 and 100%. Furthermore, three studies specifically examined the clinical utility of the Hoffmann sign in DCM diagnosis [16, 29, 30]. The Hoffmann sign demonstrated sensitivity between 58 and 62% and specificity between 78 and 86%.

Kobayashi et al. [20] reported that the 10-s grip and release test had a sensitivity of 88.2% and a specificity of 78.1%. Machino et al. [31] found that the same test had a sensitivity of 77.3% and a specificity of 58.8%. Machino et al. [31] also evaluated the 10-s step test, reporting a sensitivity of 92.3% and a specificity of 67.8%.

Two studies examining the extent of motor and sensory impairment found that motor impairment ranged from 53–75%, whilst specificity ranged from 18–64% [32, 33].

Lastly, a prospective study by Soufi et al. [34] evaluated the most discriminative clinical signs in patients with DCM. They reported that Tromner’s sign had a sensitivity of 97% and a specificity of 35%. The Hoffmann sign demonstrated a sensitivity of 89% and a specificity of 41%, while hyperreflexia of the biceps and triceps showed a sensitivity of 61% and a specificity of 74%. Tandem gait had a sensitivity of 66% and a specificity of 84%.

These findings are summarised in Table 2.

### The sensitivity and specificity of symptoms of degenerative cervical myelopathy

Cook et al. [18] reported that the most sensitive symptoms for diagnosis DCM was neck pain (94%), followed by the loss of dexterity (72%). Conversely, the specificity of these symptoms were low, with 26% for loss of dexterity and 18% for neck pain. Gait clumsiness and hand numbness were both reported to have a sensitivity of 56%. The results of Cheung et al. [35] indicated that the sensitivity of neck or shoulder pain was 76%, with a low specificity at 11%.

In a prospective study by Hori et al. [36], the findings demonstrated that numbness and pain had moderate sensitivity (61%) and specificity (60%) for diagnosing DCM, whilst other reported symptoms had low sensitivity but high specificity.

Soufi et al. [34] reported that neck pain (81%) was the most sensitive symptom for diagnosing DCM, followed by limitations in daily activities (77%). Other symptoms, such as hand incoordination (52%), altered hand sensation (76%), upper extremity weakness (51%), gait imbalance (63%), difficulty lifting heavy objects (75%), and exacerbation of neck pain during driving (70%) or reading (71%), also showed high sensitivity but varying degrees of specificity. Autonomic dysfunction had low sensitivity (24%) but high specificity (95%), making it a more specific but less sensitive indicator of DCM.

These findings are summarised in Table 2.

### Thematic analysis of diagnostic clinical signs and symptoms in degenerative cervical myelopathy

The thematic analysis of the selected studies elucidated key patterns and themes pertinent to the diagnosis of DCM (Table 3).

The Tromner sign and hyperreflexia were identified as highly sensitive clinical signs, demonstrating significant efficacy in the initial screening and identification of patients with DCM. Conversely, specific clinical signs such as the Babinski, Tromner sign, clonus, and inverted supinator sign were noted for their high specificity, making them essential for confirming the diagnosis by effectively excluding patients without DCM. However, no clear association was observed between certain clinical signs, including the Hoffmann sign, Babinski sign, and hyperreflexia, and the severity of the disease. This finding indicates that while these signs are valuable for diagnosis, they do not necessarily correlate with the severity of DCM.

Furthermore, the analysis identified hand numbness, hand paresthesias, loss of dexterity, gait abnormalities, and neck pain as highly sensitive symptoms for diagnosing DCM. Conversely, autonomic dysfunction demonstrated high specificity.

## Discussion

Establishing diagnostic criteria for DCM is invaluable for clinical practice, akin to the Wells’ Score for Pulmonary Embolus [37], as it enhances the triage of high-risk DCM patients. With an aging global population, the incidence of degenerative spinal conditions is expected to rise, underscoring the necessity for early and accurate diagnosis. The AO Spine RECODE-DCM (Research Objectives and Common Data Elements for Degenerative Cervical Myelopathy) project has identified this as a research priority [38, 39]. Given the clinical-radiological nature of DCM, there is a significant gap in clinical knowledge regarding which signs and symptoms are most predictive of the condition. Hence, this scoping review aims to outline the clinical signs and symptoms essential for inclusion in DCM diagnostic criteria. Through systematic refinement and stakeholder engagement, these evidence-based clinical recommendations will assist healthcare professionals, particularly primary care physicians, in accurately identifying DCM, even when it presents subtly. Early detection is crucial, as it significantly impacts surgical and patient outcomes.

The development of a codebook in this review represents a significant step toward improving the translation of research into clinical practice. Using thematic analysis, text excerpts from the included studies were examined to identify their relationship to the research question, with codes iteratively refined through data analysis. This process grouped similar codes into categories and broader themes, creating a structured framework for understanding diagnostic features of DCM. Stakeholder consultation played a pivotal role in shaping the codebook, providing valuable input to refine findings, address gaps in the literature, and ensure the results were both evidence-based and clinically relevant. This structured and collaborative approach bridges the gap between research and practical application, facilitating the integration of findings into clinical workflows.

Based on the results of the thematic analysis, three key themes emerged to encapsulate the diagnostic utility of the clinical signs and symptoms of DCM across the included studies. The first theme, High Sensitivity Signs, included Tromner’s sign and hyperreflexia, both identified as highly sensitive indicators useful for initial screening of DCM. The Tromner sign, an alternative clinical test to the Hoffman sign, demonstrated superiority in both sensitivity (93–97%) and specificity (35–100%) compared to the Hoffmann sign. Moreover, the Tromner sign’s diagnostic sensitivity remained high across varying degrees of myelopathy, highlighting its clinical utility [26]. While hyperreflexia is noted to be a highly sensitive sign for DCM diagnosis, its sensitivity varies by location of the lesion. Evaluation of individual reflexes demonstrated a wide range of sensitivities: biceps (18–62%), triceps (36–40%), brachioradialis (21%), patellar (22–33%), Achilles (15–26%), and suprapatellar reflex (56%).

The second theme, High Specificity Signs, encompassed the Babinski sign, Tromner sign, clonus, and the inverted supinator sign, all noted for their high specificity and value in confirming a DCM diagnosis. Among these, the Babinski sign and clonus have low sensitivity but high specificity, consistent with previous studies [18]. Though the Babinski sign was highly specific among the case-controlled studies in this review, the control groups were either healthy participants or patients with cervical spine complaints without imaging evidence of spinal cord compression or myelomalacia. Consequently, the specificity values for the Babinski sign might be falsely high, as this clinical sign can also be found in patients without cervical or spondylotic disorders causing upper motor neuron dysfunction. Additionally, variability in assessment methods and interrater reliability across different studies may impact the reported specificity values, underscoring the need for standardized diagnostic protocols in future research.

Lastly, the third theme, Symptom Sensitivity and Specificity, identified hand numbness, hand paresthesias, loss of dexterity, gait abnormalities, and neck pain as highly sensitive symptoms of DCM. Although less common, autonomic dysfunction and bladder dysfunction are noted for their high specificity in severe DCM cases. While bladder and bowel dysfunction were less frequently reported in the included studies, they serve as important markers of advanced disease and should be considered during diagnosis. Among the symptoms, it has been reported that 85% of DCM patients may present with at least one symptom involving their hands [40]. However, the specificity of neck pain is relatively low, with estimates indicating that 30–50% of adults experience neck pain annually [41]. On average, a primary care practitioner will encounter seven cases of neck-related symptoms per week, underscoring the importance of examining patients with neck pain for evidence of myelopathy [42].

Patients with DCM often present with subtle gait abnormalities, such as instability. Though not assessed in this review, it is crucial to consider recurrent falls in DCM patients, particularly the elderly [43]. This demographic may perceive gait instability as a natural aspect of aging and, consequently, may not report it when questioned about symptoms. Functional tests like tandem walk and Rhomberg sign could aid in assessing balance and coordination deficits. However, similar abnormalities can be observed in other neurodegenerative conditions, such as multiple sclerosis, Parkinson’s disease, or peripheral neuropathies, necessitating a thorough differential diagnosis [44]. Grip release tests, though reported variably, may provide additional diagnostic utility when used alongside other clinical signs and symptoms. Therefore, when taking patient history, it is essential to critically evaluate hand function and fine motor skills, gait abnormalities, neck pain, and autonomic dysfunction.

Importantly, the diagnostic accuracy of DCM may be enhanced by combining specific signs and symptoms. For instance, pairing highly sensitive signs, such as hyperreflexia and Tromner sign, with highly specific signs, such as the Babinski sign and clonus, could provide a more robust framework for diagnosis. Similarly, incorporating symptoms like hand incoordination with confirmatory signs like the inverted supinator sign could improve diagnostic confidence. Multimodal assessments, rather than evaluating signs or symptoms in isolation, are critical to achieving higher diagnostic accuracy. This combined approach underscores the need for further research into how paired or grouped findings can optimize diagnostic strategies for DCM.

Moreover, the lack of studies comparing the sensitivity and specificity of symptoms between DCM patients and healthy controls means that there is limited information on the specificity values of symptoms for DCM. This is likely due to poorly defined inclusion criteria or reliance on the absence of imaging findings, such as cervical spinal cord compression or hyperintensity on T2-weighted MRI images in these studies. Secondly, the heterogeneity in control groups, ranging from healthy individuals to those with non-myelopathic cervical spine complaints, may skew the reported sensitivity and specificity values. Nonetheless, this scoping review provides a diagnostic framework that will aid healthcare professionals in identifying DCM.

## Conclusion

Highly sensitive signs, such as the Tromner sign and hyperreflexia can be employed as screening tools for DCM. Whilst high specificity signs such as Babinski, clonus and inverted supinator sign are recommended in confirming DCM diagnosis. Symptoms like neck pain, hand incoordination, and altered hand sensation should heighten the index of suspicion and guide the clinical differential diagnosis of DCM and reduce diagnostic delays.

## Supplementary information

Supplementary Table 1. Database search strategy using Cochrane

Supplementary Table 2. Study characteristics of articles deemed eligible for inclusion by search strategy
