# Supplementary material for: Clinical signs and symptoms for degenerative cervical myelopathy: a scoping review of case-control studies to facilitate early diagnosis among healthcare professionals with stakeholder engagement
Source: Spinal Cord. 2025 Feb 26;63(3):171–80. doi: 10.1038/s41393-025-01065-1 (PMC11906348; doi:10.1038/s41393-025-01065-1)
Supplement: Supplementary file 1 — Supplementary Table 1. Database search strategy using Cochrane [file 41393_2025_1065_MOESM1_ESM.docx]

Supplementary Table 1. Database search strategy using Cochrane

| **No.** | **Query** |
| --- | --- |
| #1 | "degenerative cervical myelopathy" OR "cervical spondylotic myelopathy" |
| #2 | "signs" OR "symptoms" OR "clinical features" OR "Tromner’s sign" OR "Babinski" OR "Hoffmann" OR "Clonus" OR "Inverted supinator sign" OR "Hyperreflexia" OR "Hyperreflexia of the biceps and triceps" OR "Hyperreflexia of the biceps" OR "Hyperreflexia of the triceps" OR "Hyperreflexia at the patella" OR "Hyperreflexia at the Achilles" OR "Hyperreflexia at brachioradialis" OR "Suprapatellar reflex" OR "Absence of deep tendon reflexes" OR "Hand withdrawal reflex" OR "Gait deviation" OR "tandem gait" OR "sensory impairment" OR "motor impairment" OR "weakness and wasting of deltoid muscles" OR "weakness and wasting of shoulder girdle" OR "neck pain" OR "neck stiffness" OR "gait abnormalities" OR "gait disturbance" OR "autonomic dysfunction" OR "loss of dexterity" OR "hand numbness" OR "hand paraesthesia" OR "upper extremity weakness" OR "numbness" OR "pain" OR "tremor" OR "cervical vertigo" OR "hypalgesia" OR "jitteriness" OR "apraxia" |
| #3 | "sensitivity" OR "specificity" OR "diagnostic accuracy" OR "predictive value" OR "likelihood ratio" |
| #4 | "imaging" OR "radiology" OR "MRI" OR "CT scan" |
| #5 | #1 AND #2 AND #3 NOT #4 |
