# Supplementary material for: Clinical signs and symptoms for degenerative cervical myelopathy: a scoping review of case-control studies to facilitate early diagnosis among healthcare professionals with stakeholder engagement
Source: Spinal Cord. 2025 Feb 26;63(3):171–80. doi: 10.1038/s41393-025-01065-1 (PMC11906348; doi:10.1038/s41393-025-01065-1)
Supplement: Supplementary file 2 — Supplementary Table 2. Study characteristics of articles deemed eligible for inclusion by search strategy [file 41393_2025_1065_MOESM2_ESM.docx]

Supplementary Table 2. Study characteristics of articles deemed eligible for inclusion by search strategy

| **No.** | **Author(s)** | **Year** | **Title** | **Study Design** | **Patient Demographics** | **Symptoms/Signs** |
| --- | --- | --- | --- | --- | --- | --- |
| 1 | Archer KR, Bydon M, Khan I, et al. | 2020 | Development and Validation of Cervical Prediction Models for Patient-Reported Outcomes at 1 Year After Cervical Spine Surgery for Radiculopathy and Myelopathy | Retrospective | Cervical myelopathy group = 2641   - Mean (SD) age: 60.4 (11.4) - Men: 53% - Women: 47%   Control group = 4988   - Mean (SD) age: 54.6 (11) - Men: 50% - Women: 50% | Motor impairment |
| 2 | Cao J, Liu Y, Wang Y, et al. | 2019 | A Clinical Correlation Research of the Hoffmann Sign and Neurological Imaging Findings in Cervical Spinal Cord Compression | Retrospective | - Cervical myelopathy group = 56 - Control group = 51 | Hoffmann |
| 3 | Chaiyamongkol W, Laohawiriyakamol T, Tangtrakulwanich B, et al. | 2017 | The Significance of the Tromner Sign in Cervical Spondylotic Myelopathy Patient | Prospective | Cervical myelopathy group = 36   - Mean (SD) age: 59.9 (9.9) - Men: 77.8% - Women: 22.2%   Control group = 14   - Mean (SD) age: 53.5 (10.3) - Men: 21.4% - Women: 78.6% | - Babinski - Hoffmann - Inverted radial reflex - Tromner |
| 4 | Chang CW, Chang KY and Lin SM. | 2011 | Quantification of the Trömner signs: a sensitive marker for cervical spondylotic myelopathy | Prospective | - Cervical myelopathy group = 46 - Control group = 30 | - Hoffmann - Tromner |
| 5 | Cheung PWH, Wong CKH, Lau ST, et al. | 2018 | Psychometric Validation of the Adapted Traditional Chinese (Hong Kong) Version of the Japanese Orthopaedic Association Cervical Myelopathy Evaluation Questionnaire (JOACMEQ) | Prospective | Cervical myelopathy group = 63   - Mean (SD) age: 58.5 (12.2) - Men: 60% - Women: 40%   Control group = 37 | Neck and shoulder pain |
| 6 | Cook C, Brown C, Isaacs R, et al. | 2010 | Clustered clinical findings for diagnosis of cervical spine myelopathy | Prospective | Cervical myelopathy group = 88   - Mean (SD) age: 56.9 (12.5) - Men: 54.5% - Women: 45.4%   Control group = 161   - Mean (SD) age: 53.3 (15.6) - Men: 46.6% - Women: 52.8% | - Babinski - Clonus - Gait deviation - Hoffmann - Hyperreflexia (biceps, quadriceps, achilles) - Inverted supinator sign |
| 7 | Cook C, Roman M, Stewart KM, et al. | 2009 | Reliability and diagnostic accuracy of clinical special tests for myelopathy in patients seen for cervical dysfunction | Prospective | - Cervical myelopathy group = 18 - Control group = 27 - Mean (SD) age: 52 (13.4) - Men: 41% - Women: 59% | - Babinski - Clonus - Gait clumsiness - Hand numbness - Hand withdrawal reflex - Hoffmann - Hyperreflexia of triceps and bicep - Inverted supinator sign - Loss of dexterity - Neck pain - Suprapatellar quadriceps |
| 8 | Glaser JA, Curé JK, Bailey KL, et al. | 2001 | Cervical spinal cord compression and the Hoffmann sign | Prospective | - Cervical myelopathy group = 48 - Control group = 76 | Hoffmann |
| 9 | Grijalva RA, Hsu FP, Wycliffe ND, et al. | 2015 | Hoffmann sign: clinical correlation of neurological imaging findings in the cervical spine and brain | Retrospective | - Cervical myelopathy group = 91 - Control group = 80 | Hoffmann |
| 10 | Harrop JS, Naroji S, Maltenfort M, et al. | 2010 | Cervical myelopathy: a clinical and radiographic evaluation and correlation to cervical spondylotic myelopathy | Retrospective | Cervical myelopathy group = 54   - Mean age: 56.9 - Men: 44% - Women: 56%   Control group = 49   - Mean age: 49.5 - Men: 47% - Women: 53% | - Babinski - Cross – abductor - Gait abnormality - Hoffmann - Hyperreflexia of the upper and lower extremities - Sensory impairment |
| 11 | Hori M, Fukunaga I, Masutani Y, et al. | 2012 | New diffusion metrics for spondylotic myelopathy at an early clinical stage | Prospective | Cervical myelopathy group = 18   - Mean (SD) age: 63.3 (10.8) - Men: 33% - Women: 53%   Control group = 15   - Mean (SD) age: 50.5 (16.2) - Men: 47% - Women: 66.7% | - Apraxia - Cervical vertigo - Hypalgesia - Jitteriness - Neck stiffness - Numbness - Pain - Tremor |
| 12 | Kobayashi H, Otani K, Nikaido T, et al | 2022 | Development of a Novel Diagnostic Support Tool for Degenerative Cervical Myelopathy Combining 10-s Grip and Release Test and Grip Strength: A Pilot Study | Prospective | - Cervical myelopathy group = 247 - Control group = 721 | - Grip and release test - Grip strength |
| 13 | Machino M, Ando K, Kobayashi K, et al. | 2019 | Cut off value in each gender and decade of 10-s grip and release and 10-s step test: A comparative study between 454 patients with cervical spondylotic myelopathy and 818 healthy subjects | Prospective | - Cervical myelopathy group = 454 - Control group = 818 | Grip and release test |
| 14 | Phillips DG. | 1975 | Upper limb involvement in cervical spondylosis | Retrospective | - Cervical myelopathy group = 151 - Control group = 50 | - Absence of deep tendon reflexes - Motor deficits - Sensory impairment - Weakness and wasting of shoulder girdle and deltoid muscles |
| 15 | Rhee JM, Heflin JA, Hamasaki T, et al. | 2009 | Prevalence of physical signs in cervical myelopathy: a prospective, controlled study | Prospective | Cervical myelopathy group = 39   - Mean age: 58 - Men: 56% - Women: 44%   Control group = 37   - Mean age: 48 - Men: 49% - Women: 51% | - Babinski - Hoffmann - Hyperreflexia of biceps, triceps, brachioradialis, patella, achilles - Inverted brachioradialis - Sustained clonus |
| 16 | Soufi K, Balatbat P, Perez T, et al. | 2024 | Improving diagnosis and early detection of degenerative cervical myelopathy (DCM): a prospective cohort  study | Prospective | - Cervical myelopathy group = 130 - Control group = 82 | - 1st dorsal interosseus - Altered hand sensation - Autonomic dysfunction - Difficulty lifting heavy objects - Exacerbation of neck pain during driving or reading - Gait imbalance - GRASSP-Myelopathy test - Hand incoordination - Hoffmann - Hyperreflexia of the biceps and triceps - Impairment in gait and balance - Intrinsic hand strength deficits - Limitations in daily activities - Neck pain - Sensory impairments - Tromner - Upper extremity weakness - Weakness in thumb opposition |
